# The Growth and Reproduction of Bone

**Published:** 1873-10

**Authors:** Van S. Lindsley

**Affiliations:** Professor of Physiology in the University of Nashville.


### ARTICLE V.

### *The Growth and Reproduction of Bone.*

BY VAN S. LINDSLEY, M. D.

Professor of Physiology in the University of Nashville.

* * * * Having now examined the structure of bone, its growth and formation, we come, so far as regards the surgeon, to the practical application of our knowledge to the reproduction of bone in case of injuries.

We omit giving a detailed history of the various stages of a case of fracture, or resection, from the time of injury to the final restoration of the part to its normal condition, and will confine ourselves especially to the exact manner in which union takes place in fractured bone.

It will be admitted by all, that, when a bone is fractured, there is an effusion of some sort, which we call neoplasm, and that it comes from the surrounding structures.

What is the origin of this neoplasm, or new formation? Is it derived from the extremities of the bone itself, from the periosteum, the muscular and connective tissue? Or is the effused blood transformed into new bone, according to the old idea? Must cartilage precede the formation of bone, or may ossification occur without the intervention of cartilage?

Throwing aside all theories as to the best possible manner in which it *might* take place, let us lift the veil, so far as practicable, and actually watch with our eyes the plastic hand of nature as she prepares to weld together the dissevered bone, and make it whole and sound as the other.

Investigations prove that the new formation around a fracture occurs in the medullary and Haversian canals, in the periosteum and adjacent muscular and tendinous tissue. A large extravasion is disturbing here, as in the healing of wounds of the soft parts, for part of it must be absorbed, and the remainder organized. Among other ingredients in the effused material are small round cells, which rapidily increase, and afterward constitute the greater part of its bulk. According to the observations of Cohnheim, these cells are not newly formed, but are white blood corpuscles escaped from ruptured vessels.

It is difficult to understand the changes that now take place in the Haversian canals, subsequent to the effusion, without specimens of bone showing the changes, or faithful diagrams to illustrate them.

It must be remembered that blood-vessels occupy the Haversian canals, and that these vessels are connected to the walls of the bony canals by means of delicate areo tissue. Now what we observe is this—the cell formation exudes between the coats of the vessels and thebony wall of the canal, and forming gradually, the effect is to create pressure upon the bone partitions between the Haversian canals, and cause their absorption. If, at this stage, we macerate a bone thoroughly, so as to remove all the soft structures and vessels, it presents a corroded, roughened appearance, is more porous, and weighs less than normal bone.

Investigators, Bilroth of Vienna among them, have found difficulty in explaining why the bone, under these circumstances, is absorbed, and by what agency it takes place. "At present it is not known," says Bilroth, "how the lime salts are dissolved in this process. I think probably the new formation in the bone develops lastic acid, which changes the carbonate and phosphate of lime into soluble lactate of lime, and that this is taken up and removed by the vessels. It would also be possible for the organic bases of the bone, the so-called osseous cartilage, to be first dissolved by the inflammatory neoplasia, and then there would be a breaking down of the chalky substance, whose molecules would subsequently be removed, even if undissolved."

As the chemist and physiologist can give us no clue as to the how and why in this case, and can suggest no mode of experimenting, by which the mystery may be solved, we must, for the present, attribute it to that vital force, acting behind cell-agency, which we everywhere see in the living body, but cannot explain. Through this cell-agency, with the presence of bone to determine, by a kind of catalysis, what shall be the final result, we have the two principal factors with which to work out the problem.

This is the only instance in the body, so far as I know, in which the new formation partakes of the same grade of structure as that which was destroyed. When muscle or skin is divided, reunion takes place by means of connective tissue—a lower grade of structure. But when bone is divided, reunion is affected by the reproduction of bone itself.

Were it otherwise—did reparation of bone take place by means of cartilage, tendinous, or fibrous tissue, we would *always* have pseudarthrosis after every fracture, instead of occasionally, and the poor unfortunate individual with a broken thigh would be the most pitiable object of helplessness that could greet our sight.

Let us return to the changes going on in the Haversian canals, and the now solidifying external and internal callus. Here we find the vessels of the canals extending across from one fractured extremity to the other, and also permeating the new formation in the medullary canal, and on the surface of the bone. Now from all we gather from recent investigations, the new ossific matter is deposited around the vessels in the enlarged Haversian canals of the old bone, and takes the place of the effused cell; the Haverian canals become less and less by osseous deposit upon their walls, until they are reduced to their normal size.

In some of the smaller animals, and in the bones of young persons, the callus first become cartilaginous, and is then ossified ; but in adults ossification commences directly, without formation of cartilage.

We became convinced, some time since, that undue importance had been attributed to the periosteum by Jourdan of Manchester, and others, in the reparation of bone.

The periosteum, so far as we are able to determine, contributes its mite of assistance when present, upon the principle that the more intact the parts, the easier and quicker will be the reparation of the injury, but nothing more: there is no peculiar osteoplastic properties inherent in it, by which it takes precedence of the other tissues, presides over, and determines the process of bone-making.

In fracture, the old periosteum is involved in the mass of the external callus, and a new membrane, which afterwards becomes periosteum, is formed on the external surface. And at points of bone where tendons and ligaments are inserted, and there can be no periosteum, fractures unite and heal as firmly as elsewhere. The periosteum holds the same relation to the bone that the pia-mater does to the brain. Its chief use is evidently to support the vessels going to the bone, and afford them a bed in which they may subdivide into fine branches, and enter the dense tissue at numerous points.—*Nashville Jour. of Med. & Surg.*

